# Improving Food‐Related Inhibitory Control Through an mHealth Intervention—A Secondary Outcome Analysis of an RCT

**DOI:** 10.1002/osp4.70026

**Published:** 2024-12-02

**Authors:** Natalie Schoemann, Caroline Seiferth, Magdalena Pape, Tanja Färber, Stephan Herpertz, Sabine Steins‐Loeber, Jörg Wolstein

**Affiliations:** ^1^ Department of Psychopathology University of Bamberg Bamberg Germany; ^2^ Division of Clinical Psychology and Psychotherapy Freie Universität Berlin Berlin Germany; ^3^ Department of Clinical Psychology and Psychotherapy University of Bamberg Bamberg Germany; ^4^ Department of Psychosomatic Medicine and Psychotherapy Ruhr University Bochum Bochum Germany

**Keywords:** food‐related inhibitory control, impulsivity, mHealth, obesity

## Abstract

**Background:**

Experimental studies reveal that deficits in food‐related inhibitory control, rather than general impulsiveness, are closely linked to overweight and obesity. To date, the real‐world implications remain unknown, and it is unclear whether these results are supported in the clinical field.

**Objective:**

To examine the effectiveness of a mobile health (mHealth) intervention with cognitive and behavioral therapeutic elements in altering impulsiveness and food‐related inhibitory control.

**Methods:**

Prespecified secondary outcome analysis of a randomized controlled trial. Participants with overweight/obesity (BMI: *M* = 33.35 kg/m^2^, SD = 3.79 kg/m^2^, *N* = 213) were randomly assigned to either a 12‐week mHealth intervention (*n* = 116) or wait‐list control group (*n* = 97). The Barratt‐Impulsiveness‐Scale (BIS‐15) and the Food‐Related Inhibitory Control Scale (FRIS) were administered at baseline (T0) following the intervention (T1), at 9 and 15 month post baseline (T2, T3). Multi‐level analyses were calculated.

**Results:**

Compared to the control group, the intervention group reported higher food‐related inhibitory control on several subscales of the FRIS: In Withholding in Social Situations at T1 (95% CI: 0.06–0.46) and T2 (95%CI: 0.09–0.50), Action Cancellation at T1 (95%CI: 0.05–0.45), Resisting despite Craving at T1 (95% CI: 0.07–0.49), Withstanding Rewarding Food at T2 (95%CI: 0.08–0.55) and Action Withholding at T3 (95% CI: 0.01–0.55). No differences were found for trait impulsiveness (T1: 95%CI: −1.91–0.47; T2: 95%CI: −1.65–0.84; T3: 95%CI: −0.88–1.67).

**Conclusions:**

Food‐related inhibitory control, rather than global measures of impulsiveness, addresses the critical association between inhibitory control and health‐conscious dietary choices and can be improved by mHealth intervention.

**Trial Registration:**

ClinicalTrials.gov identifier: NCT04080193

## Graphical abstract

Deficits in food‐related inhibitory control as a component of trait impulsiveness are associated with weight status and can be influenced by cognitive and behavioral interventions. In individuals with overweight or obesity, an mHealth approach with cognitive and behavioral modules is effective in modifying food‐related inhibitory control, but not trait impulsiveness in general. Implementing interventions that target not only manifest behavioral patterns but also consider the influential role of food‐related inhibitory control may therefore facilitate sustainable modifications in weight‐related behaviors.

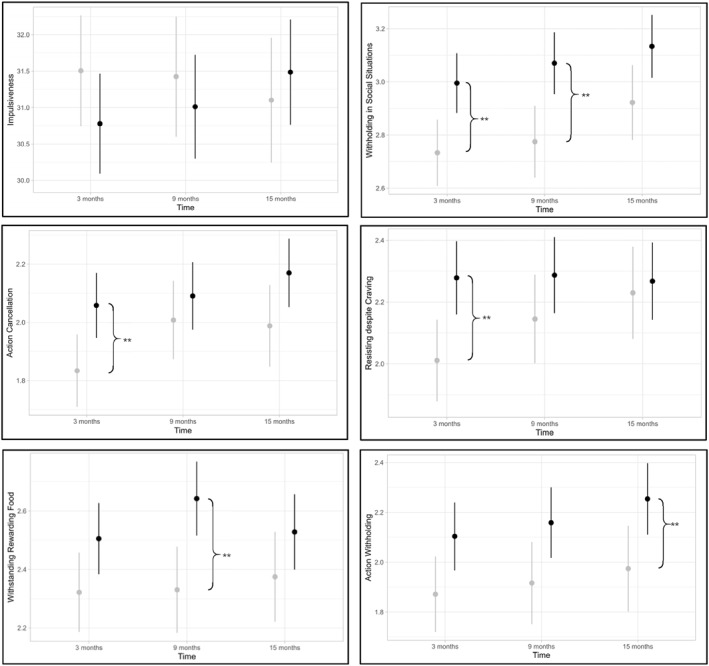

## 1 Introduction

Understanding the importance of food‐related inhibitory control has the potential to refine and optimize weight management interventions [1]. Empirical evidence indicates that a higher body mass index (BMI) is associated with reduced inhibitory control [2, 3]. Ineffective inhibitory control represents the motivational and behavioral facets inherent to trait impulsiveness. Trait impulsiveness is the general tendency to respond to urges, desires, or habits prematurely and without anticipatory foresight [2]. For example, in metabolic and bariatric surgery research, postoperative self‐reported impulse control difficulties are linked to diminished weight loss and heightened emotional distress in the early postoperative period [4]. Additionally, individuals being considered for bariatric surgery have been shown to display maladaptive or pathological eating behaviors [5]. Individuals exhibiting deficiencies in inhibitory control are prone to act without careful reflection of potential consequences [2, 6]. Some studies have found positive correlations between trait impulsiveness and weight increasing eating habits, for example emotional eating, overeating in response to negative emotional states, or making food choices based on taste, rather than long‐term health considerations. These associations were found specifically in individuals with binge eating disorder and higher negative urgency, i.e. the tendency to act impulsively when emotionally distressed [3, 7]. In addition, impulsive eating and deficits in food‐related inhibitory control have been linked to food addiction [8]. However, in other studies, trait impulsiveness did not differ between weight groups [9]. In view of the multidimensional conceptualization of impulsiveness, it can be assumed that interindividual differences in some facets may present stronger associations with BMI and eating behaviors than others. The abovementioned ambivalent findings may be ascribed to sample characteristics but at least partially also to the choice of global measures and the resulting lack of differentiation among relevant variables. For instance, in the field of alcohol addiction research, binge drinking was only predicted by an impairment of inhibition in response to alcoholic stimuli rather than general impulsiveness [10]. Overall, focusing on food‐related inhibitory control, rather than relying on global measures of impulsiveness, may provide a targeted and nuanced approach to address the critical link to health‐conscious dietary choices.

Food‐related inhibitory control subsumes action withholding and action cancellation as motoric responses linked to the desire to consume food. These include the ability to restrain from food consumption or the ability to stop eating when satiety is registered and are closely linked to factors at the individual level [1, 2, 6, 11]. Individuals with high delay discounting preferring high immediate rewards despite long‐term losses tend to consume quickly available unhealthy foods when hungry rather than prepare healthy meals [12]. Individuals with heightened reward sensitivity perceive food as rewarding and report difficulties in withstanding rewarding foods [13]. A curvilinear relationship has been observed between reward sensitivity and BMI, with a moderately positive association for individuals with a BMI between 18 and 30 and a negative correlation in individuals with a BMI higher than 30 [14]. Furthermore, food‐related inhibitory control is also associated with social contexts. Evidence suggests that individuals tend to consume more food with friends compared to when they eat alone [15]. In summary, a lack of ability to override a planned or initiated action to unhealthy food cues is associated with unfavorable weight—Enhancing eating habits such as loss of control, overeating, emotional eating, or external eating [16].

These findings demonstrate the need for psychological interventions targeting underlying food‐related cognitions and executive functioning in individuals with overweight or obesity [17]. Acceptance‐based interventions, which focus on tolerating discomfort, value‐driven behavior, and metacognitive awareness, as well as interventions focusing on changing cognitive and behavioral habits have proven to be effective in reinstating participants' weight management abilities and enhancing their food‐related inhibitory control [18, 19, 20]. Food related cognitive habits include learned associations between food and emotion, whereas behavioral habits include consuming food to cope with uncomfortable emotional states [20]. Employing interventions that address not only overt behavior patterns but also recognize the interplay with and pivotal role of underlying cognitive processes may have the potential to foster enduring changes in weight‐related behaviors.

Evidence indicates that mHealth interventions represent an effective adjunctive treatment option for overweight and obesity [21]. The smartphone based psychological mHealth application I‐GENDO provides a weight‐loss intervention with multiple evidence‐based treatment components such as self‐ and emotion regulation skills [22]. While the effectiveness of I‐GENDO regarding changes in eating behavior and BMI was demonstrated in a randomized controlled trial (RCT) [23], the primary aim of this prespecified secondary outcome analysis is the evaluation of the long‐term effectiveness of I‐GENDO regarding reductions in impulsiveness and improvement in food‐related inhibitory control as mechanisms underlying changes in body weight. Significant group differences were predicted in food‐related inhibitory control and global impulsiveness, with higher food‐related inhibitory control and lower global impulsiveness in the intervention group compared with the control group at the end of the intervention (3 months, T1), at 9 months (T2) and 15 months (T3) after baseline assessment.

## 2 Methods

This prespecified secondary outcome analysis was pre‐registered at the OSF platform (https://osf.io/wkdsh/) and forms part of the I‐GENDO project (ClinicalTrials.gov: NCT04080193). For additional information on the study procedure and the construction of the I‐GENDO intervention, see Pape et al. [22]. The intervention effectively improved the main outcomes [24].

### 2.1 Participants

The eligibility and exclusion criteria of interested individuals were verified via an online survey. Participants were included if they: (a) were 18 years or older; (b) had a BMI of 30.00–39.99 kg/m^2^ or 25.00–29.99 kg/m^2^ with weight‐related health issues or psychosocial distress; (c) had a smartphone; (d) could read, write, and speak German; and (e) were motivated to lose weight (assessed by a yes/no question). Exclusion criteria included: (a) current pregnancy, (b) recent or ongoing participation in a psychological weight loss program, (c) psychotherapeutic treatment for weight issues, (d) past or planned bariatric surgery, (e) regular use of weight‐affecting drugs, (f) untreated weight‐related health conditions, (g) current substance abuse, major depression, or suicidal thoughts, (h) binge eating disorder or bulimia nervosa according to DSM‐5 criteria, and (i) severe cognitive impairments. Individuals reporting suspected eating disorders on the Munich ED‐Quest [25] were contacted by phone, assessed through structured interviews by experienced psychologists, and referred to appropriate support services as needed.

Initially, 214 participants were randomized. One person requested deletion of data. Therefore, a total of *N* = 213 participants (male *n* = 70; female *n* = 143) were randomly assigned to either the I‐GENDO intervention (*n* = 116) or wait‐list control (*n* = 97) study arm using a computerized random number generator.

### 2.2 Study Procedure

**FIGURE 1:**
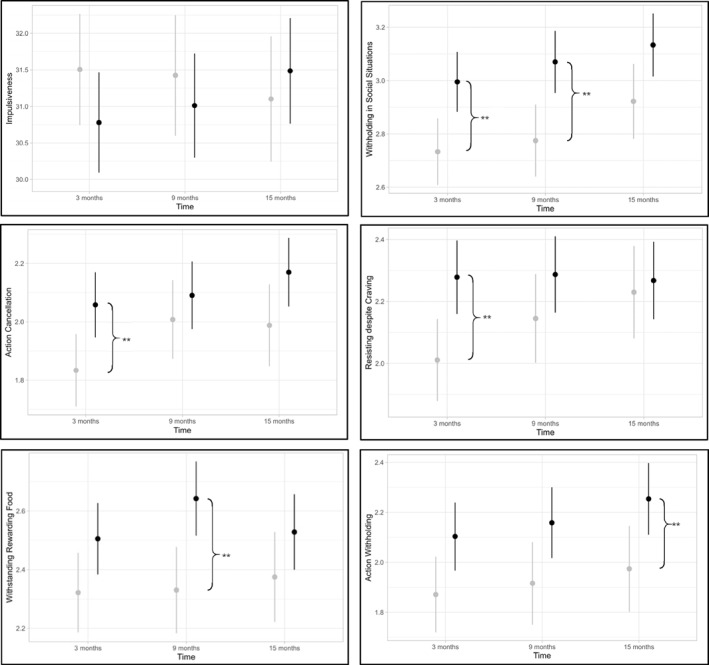
Intervention effect for each outcome at each assessment. *Description*: Between‐group differences (black = intervention group, gray = control group) adjusted for baseline values. Significant interactions are indicated by braces. *p < 0.05, **p < 0.01.

Eligible individuals completed the baseline questionnaires (T0) and were consequently invited to an in‐person briefing on the I‐GENDO study procedure at the study sites (Bochum or Bamberg, Germany) by study staff blinded to their group allocation. Participants then installed the I‐GENDO application on their smartphones (iOS or Android operating systems). The I‐GENDO app interface informed everyone about their allocation to either the intervention or control group after baseline assessment. After the 12‐week intervention phase, participants completed another questionnaire (T1). Additionally, follow‐up questionnaires were answered at 9 (T2) and 15 months (T3) post baseline (Figure 1).

## 3 Intervention

The 12‐week mHealth intervention offered seven modules addressing behavior change skills, which are related to weight‐loss management. The seven app modules targeted goal setting and motivation (introduction), stress management skills, emotion regulation skills, dealing with consequences of overweight, self‐efficacy, self‐regulation skills and relapse prevention (conclusion). I‐GENDO furthermore contained exercises targeting impulsivity and training food‐related inhibitory control such as go‐no‐go tasks with healthy and unhealthy food cues. The presentation of modules and assignment of such was tailored through self‐ and computer‐based features for each participant to enhance the efficacy of the intervention [22]. The I‐GENDO app also provided optional features such as self‐monitoring, homework sessions and a toolbox to save favored topics.

### 3.1 Measures

Self‐reported demographic information (age, gender) was collected at T0 and anthropometry (i.e., weight) was reported at all four assessments.

#### 3.1.1 Impulsiveness

For the assessment of impulsiveness, participants rated statements on personal impulsiveness on a 4‐point Likert scale ranging from 1 (rarely/never) to 4 (almost always/always) using the 15‐item German Barratt Impulsiveness Scale ‐ Short Version (BIS‐15; 25). Sum scores were calculated, with higher scores indicating higher trait impulsiveness. The questionnaire demonstrated good internal consistency (Cronbach's *α* = 0.80) and was answered at all four assessments.

#### 3.1.2 Food‐Related Inhibitory Control

The Food‐Related Inhibitory Control Scale (FRIS, C. Seiferth and colleagues, not yet published) is a newly developed questionnaire, currently revised and validated. The questionnaire initially consisted of 40 items, which were theoretically based on different inhibitory control facets. An exploratory analysis revealed a five‐factor structure with a total of 30 remaining items used for further statistical analyses (see Supporting Information S1 for additional material). Scores on the five subscales represent the following abilities: to not consume food in social situations, during which food is offered (Withholding in Social Situations); to stop eating when satiety is registered (Action Cancellation); to not immediately engage in excessive consumption of food even when a strong craving is present (Resisting despite Craving); to withstand one's perception and anticipation of food being rewarding and consuming food to recompense oneself (Withstanding Rewarding Food) and to resist the urge to eat when appealing foods or advertisements of such are perceived (Action Withholding). The subscales found are in line with the initial theoretical conceptualization, with the addition of the Withholding in Social Situations subscale. Internal consistencies ranged between *α* = 0.66 (Action Withholding) and *α* = 0.86 (Action Cancellation). See Supporting Information S1 for internal consistencies, factor loadings, items included, and items excluded (Supporting Information S1: Tables S1–S4).

Participants rated statements regarding personal food‐related inhibitory control and food‐related attitudes ranging from 0 (strongly disagree) to 5 (strongly agree). Mean scores on all subscales were calculated, with higher values indicating higher food‐related inhibitory control while lower scores point to lower food‐related inhibitory control. FRIS was answered at all four assessments.

### 3.2 Statistical Analyses

Analyses were performed using different packages of R (R Core Team, 2023) and Rstudio (Posit team, 2023). Frequencies and percentages were calculated for categorial variables, whereas means and standard deviations were used as descriptive measures for continuous variables. Socio‐demographic baseline differences between groups and for each gender within each group were tested using chi‐square distributions for categorial variables and analyses of variance (ANOVA) for continuous variables.

Baseline adjusted linear multilevel regression models (maximum likelihood estimation) were carried out in a stepwise procedure to analyze the effect of group (I‐GENDO or wait‐list control group), time (T0, T1, T2, T3), gender and possible interactions on trait impulsiveness (BIS‐15) and the subscales of the FRIS. The data follows a two‐level structure, where repeated measurements (within) are located on level 1, and group (between) on level 2. In all six separate models, participants were inserted as random intercepts, while group, time and gender were inserted as fixed factors to control for their effects. Additionally, all models were adjusted for the grand mean centered baseline value of the given outcome variable. *A p* < 0.05 was considered statistically significant.

### 3.3 Ethics

The Ruhr‐University Bochum Institutional Review Board (No. 18–6415) and the ethics committee at the University of Bamberg approved this study. All participants gave written informed consent before study participation.

## 4 Results

### 4.1 Baseline Characteristics

The average BMI at the initial measurement point was *M* = 33.35 kg/m^2^ (SD = 3.79 kg/m^2^). Baseline demographics and descriptive statistics for the BIS and FRIS subscales arranged by group are depicted in Table 1. Intervention and control groups did not differ in sociodemographic variables, with the following exception: Women in the control group were significantly younger than men in the control group (*F*(1, 95) = 6.67, *p* < 0.05, partial *η* ^2^ = 0.066). Additionally, significantly more individuals in the intervention group (*n* = 87) completed all four assessments than in the control group (*n* = 56; *χ* ^2^(1) = 6.38, *p* < 0.05).

**TABLE 1:** Baseline characteristics.

Variables	Overall		Control		Intervention	
	Control (*n* = 97)	Intervention (*n* = 116)	Female (*n* = 66)	Male (*n* = 31)	Female (*n* = 77)	Male (*n* = 29)
Demographics						
Age (in years); *M* (SD)	45.45 (12.66)	47.28 (11.65)	**43.24 (12.86)**	**50.16 (11.00)**	46.40 (12.22)	49.00 (10.38)
High school degree; *n* (%)	25 (25)	36 (31)	17 (26)	8 (26)	25 (32)	11 (28)
Married or living with a partner; *n* (%)	79 (81)	91 (78)	52 (79)	27 (87)	57 (74)	34 (87)
Impulsiveness; *M* (SD)	30.89 (5.61)	30.82 (6.25)	30.80 (5.73)	31.06 (5.42)	30.39 (5.91)	31.67 (6.88)
Food‐related inhibitory control						
Withholding in social situations; *M* (SD)	2.76 (0.93)	2.76 (0.91)	2.73 (0.95)	2.82 (0.89)	2.75 (0.90)	2.79 (0.95)
Action cancellation; *M* (SD)	1.90 (0.83)	1.79 (0.82)	1.86 (0.85)	1.99 (0.78)	1.74 (0.82)	1.90 (0.81)
Resisting despite craving; *M* (SD)	2.00 (0.90)	1.94 (0.85)	**1.82 (0.91)**	**2.37 (0.74)**	**1.79 (0.79)**	**2.23 (0.92)**
Withstanding rewarding food; *M* (SD)	2.42 (1.10)	2.41 (1.13)	2.32 (1.08)	2.65 (1.13)	**2.23 (1.12)**	**2.76 (1.07)**
Action withholding; *M* (SD)	1.97 (0.97)	1.96 (1.01)	**1.77 (0.90)**	**2.38 (1.01)**	**1.80 (1.00)**	**2.28 (0.95)**

*Note:* Bold text indicates significant gender differences within each group.

Furthermore, analysis of variance showed no significant baseline group differences for either the BIS‐15 sum score or the FRIS subscales. However, there were gender differences. Women reported significantly lower scores in both the intervention and control group for the Resisting despite Craving subscale (intervention group: *F*(1, 114) = 7.01, *p* < 0.01, partial *η* ^2^ = 0.057; control group: *F*(1, 95) = 8.53, *p* < 0.01, partial *η* ^2^ = 0.082) and Action Withholding (intervention group: *F*(1, 114) = 6.16, *p* < 0.05, partial *η* ^2^ = 0.051; control group: *F*(1, 95) = 8.89, *p* < 0.01, partial *η* ^2^ = 0.085). Additionally, these gender differences were also found in the intervention group for Withstanding Rewarding Food (*F*(1, 114) = 6.11, *p* < 0.05, partial *η* ^2^ = 0.051).

### 4.2 Multilevel Analyses

Model‐estimated means, standard errors, 95% confidence intervals (CI) and between‐group differences for the self‐reported outcomes adjusted for baseline value from fitted maximum likelihood repeated measures mixed models at T1, T2, and T3 are shown in Table 2. During the stepwise selection process, neither the interaction terms including gender nor the gender term itself was retained in the final model due to their lack of statistical significance. Consequently, the final models did not include these terms.

**TABLE 2:** Model‐estimated means, standard errors, and 95% CI for all outcomes at 3, 9, and 15 months.

3 months							9 months							15 months						
Control			Intervention			Between‐group difference [95% CI]	Control			Intervention			Between‐group difference [95% CI]	Control			Intervention			Between‐group difference [95% CI]
Mean	SE	95% CI	Mean	SE	95% CI		Mean	SE	95%CI	Mean	SE	95%CI		Mean	SE	95%CI	Mean	SE	95%CI	
Impulsiveness																				
31.50	0.39	[30.74, 32.27]	30.78	0.35	[30.09, 31.46]	−0.72 [−1.91, 0.47]	31.43	0.42	[30.60, 32.25]	31.01	0.36	[30.30, 31.72]	−0.41 [−1.65, 0.84]	31.10	0.44	[30.24, 31.96]	31.49	0.37	[30.77, 32.21]	0.39 [−0.88, 1.67]
Withholding in social situations																				
2.73	0.06	[2.61, 2.86]	3.00	0.06	[2.88, 3.11]	**0.26 [0.06, 0.46]**	2.77	0.07	[2.64, 2.91]	3.07	0.06	[2.95, 3.19]	**0.29 [0.09, 0.50]**	2.92	0.07	[2.78, 3.06]	3.13	0.06	[3.02, 3.25]	0.21 [−0.00, 0.46]
Action cancellation																				
1.83	0.06	[1.71, 1.96]	2.06	0.06	[1.95, 2.17]	**0.23 [0.05, 0.45]**	2.01	0.07	[1.87, 2.14]	2.09	0.06	[1.97, 2.21]	0.08 [−0.10, 0.32]	1.99	0.07	[1.85, 2.13]	2.17	0.06	[2.05, 2.29]	0.18 [−0.01, 0.42]
Resisting despite craving																				
2.01	0.07	[1.88, 2.14]	2.28	0.06	[2.16, 2.40]	**0.28 [0.07, 0.49]**	2.15	0.07	[2.00, 2.29]	2.29	0.06	[2.16, 2.41]	0.15 [−0.07, 0.38]	2.23	0.08	[2.08, 2.38]	2.27	0.06	[2.14, 2.39]	0.05 [−0.18, 0.28]
Withstanding rewarding food																				
2.32	0.07	[2.19, 2.46]	2.51	0.06	[2.38, 2.63]	0.19 [−0.04, 0.41]	2.33	0.08	[2.18, 2.48]	2.64	0.06	[2.52, 2.77]	**0.32 [0.08, 0.55]**	2.37	0.08	[2.22, 2.53]	2.53	0.07	[2.40, 2.66]	0.16 [−0.08, 0.39]
Action withholding																				
1.87	0.08	[1.72, 2.02]	2.10	0.07	[1.97, 2.24]	0.23 [−0.02, 0.49]	1.92	0.08	[1.75, 2.08]	2.16	0.07	[2.02, 2.30]	0.24 [−0.02, 0.51]	1.97	0.09	[1.80, 2.15]	2.25	0.07	[2.11, 2.40]	**0.28 [0.01, 0.55]**

*Note:* Bold text indicates significant between‐group effects. Displayed are the results of the multilevel model analysis for each outcome (adjusted for baseline value) and assessment (3 months, 9 months, 15 months). Each model contained an interaction term for time*group.

No significant group differences were found in global impulsiveness at any given time point between the intervention and the control groups (T1: *β* = −0.11, *p* = 0.24, difference T1: −0.72, 95% CI: −1.91–0.47; T2: *β* = −0.06, *p* = 0.53, difference T2: −0.41, 95% CI: −1.65–0.84; T3: *β* = 0.06, *p* = 0.55, difference T3: 0.39, 95% CI: −0.88–1.67).

Regarding the FRIS, participants in the intervention group reported significantly higher Withholding in Social Situations at T1 (*β* = 0.29, *p* = 0.01, difference T1: 0.26, 95% CI: 0.06–0.46) and T2 (*β* = 0.32, *p* = 0.01, difference T2: 0.29, 95% CI: 0.09–0.50) after baseline assessment. No significant group differences were found at T3 (*β* = 0.23, *p* = 0.05, difference T3: 0.21, 95% CI: −0.00–0.46).

Individuals in the intervention group reported significantly higher levels of Action Cancellation immediately after the I‐GENDO intervention compared with the control group (*β* = 0.29, *p* = 0.02, difference T1: 0.23, 95% CI: 0.05–0.45). No effect of the intervention on Action Cancellation was detected at either of the two follow‐up assessments (T2: *β* = 0.13, *p* = 0.31, difference T2: 0.08, 95% CI: −0.10–0.32; T3: *β* = 0.24, *p* = 0.06, difference T3: 0.18 95% CI: −0.01–0.42).

For Resisting despite Craving, group differences were only found immediately after the intervention at T1, with reports of higher inhibitory control in the intervention group (*β* = 0.30, *p* = 0.01, difference T1: 0.28, 95% CI: 0.07–0.49). Groups did not differ at T2 (*β* = 0.17, *p* = 0.18, difference T2: 0.15, 95% CI: −0.07–0.38) or T3 (*β* = 0.05, *p* = 0.67, difference T3: 0.05, 95% CI: −0.18–0.28).

The intervention did not influence Withstanding Rewarding Food at T1 (*β* = 0.17, *p* = 0.10, difference T1: 0.19 95% CI: −0.04–0.41). Compared to the control group, participants in the intervention group reported significantly higher inhibitory control toward rewarding foods at T2 (T2: *β* = 0.29, *p* = 0.01, difference T2: 0.32, 95% CI: 0.08–0.55). No effect of the intervention on reward sensitivity was observed at T3 (*β* = 0.15, *p* = 0.20, difference T3: 0.16 95% CI: −0.08–0.39).

Lastly, no group differences were found at T1 (*β* = 0.23, *p* = 0.07 difference T1: 0.23, 95% CI: −0.02–0.49) or T2 (*β* = 0.24, *p* = 0.07 difference T2: 0.24, 95% CI: −0.02–0.51) regarding the Action Withholding subscale. At T3, individuals in the intervention group reported significantly higher Action Withholding than participants in the control group (*β* = 0.28, *p* = 0.04 difference T3: 0.28, 95% CI: 0.0–0.55). The differences for each outcome at each assessment are illustrated in Figure 1.

## 5 Discussion

The aim of this prespecified secondary outcome analysis was to evaluate the long‐term effectiveness of the mHealth intervention I‐GENDO in altering impulsiveness and, more specifically, food‐related inhibitory control in individuals with overweight or obesity. The results show that the I‐GENDO intervention effectively improves the food‐related aspects of inhibitory control in individuals with overweight or obesity either short‐ or long‐term.

Although an effect was found in food‐related inhibitory control, no intervention effect was detected for self‐reported trait impulsiveness. As mentioned before, similar findings have been reported in the field of alcohol addiction research. For instance, binge drinking was only predicted by an impairment of inhibition in response to alcoholic stimuli rather than trait impulsiveness [10]. Furthermore, obesity research recommends investigating sub‐domains of impulsiveness related to eating behavior. The findings indicated that elevated trait impulsiveness was only observed in individuals with obesity and binge‐eating disorder. In contrast, individuals with obesity, yet without binge‐eating disorder, only displayed increased impulsiveness related specifically to food [1]. Similarly, in our study, the participants with overweight and obesity showed proximity to average values in trait impulsiveness at baseline [26]. In fact, individuals with binge‐eating disorder did not meet the inclusion criteria in our study [22]. These findings substantiate the necessity for the implementation of more specific measures of impulsiveness to detect self‐reported variations inherent in impulsive behaviors related to food. For this purpose, the FRIS with its five subscales Action Cancellation, Withholding in Social Situations, Resisting despite Craving, Withstanding Rewarding Food and Action Withholding was applied.

The study revealed meaningful group differences in food‐related Action Cancellation immediately following the intervention. There were no group differences in Action Cancellation at the follow‐up assessments. For Action Cancellation as interrupting an already initiated action, high amounts of cognitive control and inhibitory capacities are necessary [2]. It is possible that over time, motivation, and cognitive resources began to deplete and thus Action Cancellation became more difficult. A meta‐analysis concluded that specific behavioral inhibitory control training can be effective although its longevity is still unclear [27, 28].

As expected, participants in the intervention group reported meaningful improvement in food‐related inhibitory control in social situations at three and 9 months after baseline assessment compared with individuals in the control group. No group differences were found at the last follow‐up assessment. This result provides support for the short‐ and long‐term effectiveness of a cognitive and behavioral mHealth intervention in altering self‐reported inhibitory control in social situations where food is offered. Possibly, participants in the intervention group were more aware of their eating behavior in social situations due to the usage of the I‐GENDO app, which includes modules such as training sessions for dietary self‐observation (e.g. guided eating meditations and integration of mindfulness in everyday life [24]).

Participants in the intervention group reported improvement in food‐related resisting despite craving immediately after the intervention, but no differences were found at either follow‐up assessment. As mentioned before and since Resisting despite Craving is a motivational component of inhibitory control, it could be argued that immediately after the I‐GENDO intervention, motivation for behavior change was greater at the beginning than later. Individuals in weight loss programs tend to have unrealistic expectations on the amount of possible weight loss over time [29]. This could potentially lead to diminished motivation over the course of the intervention, instigated by a perceived dissonance between anticipated outcomes and actual achievement.

Group differences for Withstanding Rewarding Food were only found 9 months after baseline assessment. Contrary to expectations, no group differences were found directly after the intervention or at the last follow‐up. Previous studies have identified a moderate correlation of reward sensitivity in people with normal or overweight, whereas individuals with obesity did not reveal positive associations [14]. Since we included participants with overweight as well as obesity, results remain inconclusive. Additionally, in the I‐GENDO study, satiety was not assessed before filling out the questionnaires, even though the hunger and reward systems are closely linked [30]. This may limit the accuracy of these findings. Lastly, reward sensitivity is associated with eating behaviors such as binge or emotional eating and palatable food intake. However, contrary to the association of reward sensitivity and eating behaviors, findings related to weight status were less consistent [31]. A more detailed investigation of the ability to withstand rewarding foods and its link to eating behavior and weight is warranted to disentangle these relationships.

At the last follow‐up assessment, compared to the control group, the intervention group reported higher inhibitory control in self‐reported Action Withholding as the ability to not subdue to the urge to eat when appealing foods or advertisements are perceived. No group differences were found in either of the previous assessments. However, this finding must be interpreted with caution since this subscale did not provide sufficient internal consistency (Cronbach's *α* = 0.66).

Several limitations of the current study should be considered. Compared to the intervention group, higher dropout rates were found in the control group. Therefore, instead of analyses of variance, multilevel models were calculated to provide statistically adequate analyses that are robust to missing data [23]. Additionally, as the FRIS has not been validated in other groups, results must be interpreted with caution. Furthermore, the BIS‐15 and the FRIS employed in this study are self‐reporting measures and findings can potentially be biased. Yet, regarding the BIS‐15, a good convergent validity was found [26]. Thus, it is assumed that self‐reporting measures are also valid in assessing food‐related inhibitory control. The data of this study was collected from December 2019 to December 2021, with the COVID‐19 pandemic possibly having an impact on both intervention and control groups regarding inhibitory control and eating behavior as survey data demonstrates changes in eating behavior due to social isolation during the first COVID‐19 lockdown in spring 2020 in Germany [32]. More specifically, individuals with overweight or obesity such as the target group in this study had higher odds of changing their dietary behaviors. Nevertheless, these limitations are alleviated by the fact that both groups were equally exposed to the COVID‐19 pandemic regulations. Lastly, evidence indicates that the anticipation of future intervention among wait‐list controls such as receiving the I‐GENDO app in this study may inadvertently spur undesired improvements toward the end of the study. This might result in a potential underestimation of the intervention groups' efficacy [33].

## 6 Conclusion

To the best of our knowledge, the newly developed FRIS is the first instrument to collect self‐reported data on food‐related inhibitory control. Using this concept, rather than relying on global measures of impulsiveness, provides a nuanced approach that addresses the critical link between inhibitory control and health‐conscious dietary choices. Effective executive function of food‐related inhibitory control enables individuals to consciously regulate their eating habits in a highly obesogenic environment. Additionally, the I‐GENDO intervention significantly improved participants' behavioral and motivational aspects of food‐related inhibitory control. Thus, an mHealth approach may be effective in modifying food‐related inhibitory control in the short‐term and, in some respects, also in the long‐term.

## Conflicts of Interest

The authors declare no conflicts of interest.

## Supporting information

Supporting Information S1
