# Supplementary material for: Improving Food‐Related Inhibitory Control Through an mHealth Intervention—A Secondary Outcome Analysis of an RCT
Source: Obes Sci Pract. 2024 Dec 2;10(6):e70026. doi: 10.1002/osp4.70026 (PMC11609755; doi:10.1002/osp4.70026)
Supplement: Supplementary file 1 — Supporting Information S1 [file OSP4-10-e70026-s001.pdf]

SUPPORTING INFORMATION

TITLE: Improving food-related inhibitory control through an mHealth intervention – a secondary outcome analysis of an RCT

AUTHORS: Natalie Schoemann[1], Caroline Seiferth[1,2], Magdalena Pape[3], Tanja Färber[1], Stefan Herpertz[3], Sabine Steins-Loeber[4] & Jörg Wolstein[1]

AFFILIATION:

[1]Department of Psychopathology, University of Bamberg, Bamberg, Germany

[2]Division of Clinical Psychology and Psychotherapy, Freie Universität Berlin, Berlin, Germany

[3]Department of Psychosomatic Medicine and Psychotherapy, LWL-University Hospital, Ruhr University Bochum, Bochum, Germany

[4]Department of Clinical Psychology and Psychotherapy, University of Bamberg, Bamberg, Germany

CONTACT INFO:

E-Mail: natalie.schoemann@uni.bamberg.de

Mailing Adress:

Otto-Friedrich-Universität Bamberg

z.H. Natalie Schoemann; Professur für Pathopsychologie

Markusstr. 8a

96045 Bamberg

GERMANY

Exploratory Factor Analysis of the Food-related inhibitory Control Scale (FRIS)

Method

According to Costello and Osborne (1), a minimum 5:1 subjects-to-item ratio is necessary for exploratory factor analysis. Our sample size of $N = 213$ provided a given ratio of 5.33:1 for the recently developed 40-item FRIS. Furthermore, both Bartlett's Test of Sphericity (χ^2 (780) = 3724, $p < 0.001$) and Kaiser-Meyer-Olkin Measure of Sampling Adequacy (MSA= 0.86) indicated that data were suitable for an exploratory factor analysis. The exploratory factor analysis was calculated with Principal Component Analysis as extraction, and Promax as oblique rotation method. The number of factors was determined using scree plot, parallel analysis, and the Velicer's Minimum Average Partial (MAP) test. Internal consistency of factors was assessed with Cronbach's alpha.

Participants rated statements regarding personal food-related inhibitory control and food-related attitudes ranging from 0 (strongly disagree) to 5 (strongly agree). Mean scores on all subscales were calculated, with higher subscale values indicating higher food-related inhibitory control while lower scores point to lower food-related inhibitory control. The FRIS was answered at all four assessments.

Results

Scree plot, parallel analysis as well as the MAP test (averaged squared partial correlation: component 1 = 0.087, component 2 = 0.021, component 3 = 0.019, component 4 = 0.018, component 5 = 0.017, component 6 = 0.016) all suggested an extraction of five factors, explaining 55% of variance. A total of 10 cross-or low-loading (<.30) items was dropped to reduce the number of items and increase internal consistency. This resulted in 5 subscales. The first factor contained items describing social situations, during which one is offered food. The second factor included items describing the ability to stop eating, when satiety is registered, whereas the third factor comprised items expressing the ability to not immediately engage in excessive consumption, even when a strong craving is present. The fourth factor referred to the

perception and anticipation of food being rewarding and, even more so, consuming food to recompense oneself. The fifth factor contained items which convey the ability to resist the urge to eat when appealing snacks or advertisements are present and perceived. Subscales were termed action withholding, action cancellation, delay discounting, reward sensitivity and snacking, respectively. Internal consistencies ranged between α=0.66 and α= 0.86 (Table S1).

Table S1
Internal consistencies of and correlation between the five subscales of the FRIS and the BIS-15 at baseline assessment

n = 213	Range	1	2	3	4	5	6
1. Withholding in Social Situations	0.00-5.00	(α = 0.84)	0.54*	0.46*	0.54*	0.44*	0.00
2. Action Cancellation	0.00-5.00	-	(α = 0.86)	0.47*	0.46*	0.46*	-0.08
3. Resisting despite Craving	0.00-5.00	-	-	(α = .78)	0.51*	0.40*	0.00
4. Withstanding Rewarding Food	0.00-5.00	-	-	-	(α =0.81)	0.44*	0.12
5. Action Withholding	0.00-5.00	-	-	-	-	(α = 0.66)	-0.07
6. Impulsiveness	1.00-4.00	-	-	-	-	-	(α = 0.80)

**p ≤ .001*

Factor loadings are shown in table S2. The final 30 items and subscales are listed in table S3. The excluded items are listed in table S4.

Table S2. Factor loadings

	FRIS items	Factor loadings 1	2	3	4	5	h2	u2	com
Factor 1 (Action Cancellation)									
FRIS_17	Auch wenn mir etwas schmeckt, höre ich auf zu essen, wenn ich satt bin. (*Even if I like something, I stop eating when I'm full.*)	**0,72**	0,13	-0,01	0,05	-0,05	0,64	0,36	1,1
FRIS_18**	Bei meinen Lieblingsgerichten esse ich über meinen Hunger hinaus. (*With my favorite dishes, I eat beyond my hunger.)*	**0,41**	0,37	0,22	0,04	-0,24	0,62	0,38	3,2
FRIS_19	Sobald ich merke, dass ich satt bin, beende ich mein Essen. (*As soon as I realize that I'm full, I finish my meal.*)	**0,86**	0,03	-0,07	-0,09	0,12	0,72	0,28	1,1
FRIS_20	Wenn ich während des Essens merke, dass ich schon satt bin, höre ich auf zu essen. (*If I realize while eating that I am already full, I stop eating.)*	**0,82**	-0,08	-0,08	0,01	0,05	0,61	0,39	1
FRIS_21	Ich nehme mir nur dann eine weitere Portion, wenn ich noch nicht satt bin. (*I only eat another portion if I'm not full yet.)*	**0,76**	0,03	0,02	-0,05	0,15	0,65	0,35	1,1
FRIS_24	Wenn ich mir vornehme, nur wenig zu essen, halte ich das auch ein. (*If I resolve to eat only a little, I stick to it.*)	**0,65**	-0,07	0,09	-0,13	0,26	0,52	0,48	1,5
FRIS_30	Wenn Essen verfügbar ist, fällt es mir leicht, das Essen über mehrere Portionen oder Mahlzeiten einzuteilen. (*If food is available, I find it easy to divide the food into several portions or meals.*)	**0,51**	-0,08	0,04	0,02	0,26	0,38	0,62	1,6
Factor 2 (Withholding in Social Situations)									
FRIS_1**	In Situationen, in denen viel Essen verfügbar ist (z. B. Buffet, Feierlichkeiten, Grillen) greife ich zu, obwohl ich satt bin. (*In situations where there is a lot of food (e.g. buffets, parties, barbecues), I help myself even though I am full.)*	0,23	**0,7**	0,04	-0,04	-0,08	0,67	0,33	1,2
FRIS_3**	Wenn ich mit Menschen zusammen bin, die etwas essen, esse ich mit, ohne darüber nachzudenken. (*When I'm around people who are eating, I eat without thinking about it.*)	0,03	**0,64**	0,11	-0,09	0,3	0,59	0,41	1,5
FRIS_4**	Wenn ich eine Person sehe, die etwas isst, habe ich das Bedürfnis etwas zu essen und tue dies dann auch. (*If I see someone eating something, I feel the need to eat and then do so.)*	-0,27	**0,67**	-0,03	-0,02	0,5	0,64	0,36	2,2
FRIS_5**	Wenn mir eine andere Person etwas zu essen anbietet, was mir schmeckt, greife ich direkt zu. (*If someone offers me something to eat that I like, I take it straight away.)*	-0,1	**0,74**	0,19	-0,03	-0,03	0,59	0,41	1,2
FRIS_9**	Wenn andere etwas von ihrem Essen übriglassen, esse ich die Reste auf, obwohl ich satt bin. (*If others leave some of their food, I eat the leftovers even though I'm full.*)	0,05	**0,5**	-0,25	0,11	0,22	0,34	0,66	2,1
FRIS_22**	In Situationen, in denen viel Essen zur Verfügung steht (z. B. Buffet, Grillen, Feierlichkeiten), esse ich weiter, obwohl ich schon satt bin. (*In situations where there is a lot of food (e.g. buffet, barbecue, party), I continue to eat even though I am already full.*)	0,33	**0,65**	0,05	-0,05	-0,12	0,68	0,32	1,6
FRIS_27**	Solange andere noch essen, kann ich nicht aufhören zu essen, obwohl ich satt bin. (*As long as others are still eating, I can't stop eating even though I'm full.*)	-0,12	**0,74**	-0,28	0,06	0,38	0,62	0,38	1,9
Factor 3 (Resisting despite Craving)									
FRIS_10**	Ich esse unbewusst zwischen den Mahlzeiten. (*I eat unconsciously between meals*.)	-0,13	0,25	**0,5**	-0,26	0,44	0,56	0,44	3,2
FRIS_12**	Obwohl ich bestimmte Lebensmittel nicht essen will, kann ich ihnen dennoch nicht widerstehen. (*Even if I don't want to eat certain foods, I can't resist them.*)	0,12	-0,12	**0,5**	0,01	0,29	0,43	0,57	1,9
FRIS_14**	Wenn ich Heißhunger auf ein bestimmtes Essen verspüre, kann ich nicht widerstehen und greife zu. (*If I have a craving for a certain food, I can't resist and reach for it.*)	-0,06	0,26	**0,59**	0,03	-0,05	0,49	0,51	1,4
FRIS_15**	Wenn ich Lust auf etwas habe, esse ich es. (*When I crave something, I eat it.*)	-0,01	0,14	**0,6**	-0,07	-0,07	0,38	0,62	1,2
FRIS_23**	Ich nasche bis die angefangene Packung (z. B. Schokolade, Chips, Gummibärchen oder ähnliches) aufgebraucht ist. (*I snack until the packet I started (e.g. chocolate, potato chips, jelly babies or similar) has been used up.)*	0,04	0	**0,48**	0,27	-0,04	0,4	0,6	1,6
FRIS_31**	Wenn ich Hunger habe, wähle ich Nahrungsmittel, die sehr wenig Zubereitung erfordern, damit ich schnell essen kann. (*When I'm hungry, I choose foods that require little preparation so that I can eat quickly*.)	-0,08	-0,02	**0,73**	0,01	0,03	0,5	0,5	1
FRIS_33**	Wenn ich hungrig bin, wähle ich schnell verfügbares Essen. (*When I am hungry, I choose food that is readily available.*)	0,02	-0,18	**0,82**	0,13	-0,05	0,66	0,34	1,2
Factor 4 (Withstanding Rewarding Foods)									
FRIS_34**	Essen stellt eine Belohnung für mich dar. (*Food is a reward for me.*)	-0,1	-0,11	0,09	**0,9**	0,18	0,8	0,2	1,2
FRIS_35**	Die Erwartung an Essen stimmt mich sehr glücklich. (*The anticipation of food makes me very happy.*)	0,15	0,22	-0,15	**0,64**	-0,02	0,64	0,36	1,5
FRIS_36**	Ich belohne mich mit Essen. (*I reward myself with food.*)	-0,09	-0,06	0,12	**0,83**	0,21	0,76	0,24	1,2
FRIS_37**	Wenn ich esse, fühle ich mich gut. (*When I eat, I feel good.*)	0,02	0,24	0,02	**0,52**	-0,24	0,48	0,52	1,8
FRIS_38**	Ich bevorzuge Nahrungsmittel, die mich schnell belohnen. (*I prefer foods that reward me quickly.*)	-0,1	-0,08	0,3	**0,53**	0,25	0,5	0,5	2,2
Factor 5 (Action Withholding)									
FRIS_6	Der Anblick von Nahrungsmitteln (z. B. im Fernsehen, auf Werbeplakaten, in Zeitschriften oder ähnliches) verleitet mich nicht zum Essen. (*The sight of food (e.g. on television, on advertising posters, in magazines or similar) does not tempt me to eat.*)	0,16	0,09	-0,09	0,11	**0,64**	0,53	0,47	1,3
FRIS_7	Der Anblick von Nahrungsmitteln (z. B. im Supermarkt, beim Bäcker, auf Märkten, in Geschäften oder ähnliches) verleitet mich nicht zum Essen. (*The sight of food (e.g. in the supermarket, at the bakery, at markets, in stores or similar) does not tempt me to eat.*)	0,1	0,11	-0,12	0,28	**0,5**	0,43	0,57	1,9
FRIS_8	Auch wenn ich Gelegenheit zum Naschen habe (z. B. beim Kochen, Backen oder ähnliches), tue ich dies nicht. (*Even if I have the opportunity to snack (e.g. when cooking, baking or similar), I do not do so.*)	0,13	0,01	0	0,07	**0,41**	0,23	0,77	1,3
FRIS_13	Wenn ich nicht naschen will, mache ich es auch nicht. (*If I don't want to snack, I don't do it either.*)	0,17	-0,06	0,09	0,07	**0,54**	0,41	0,59	1,3

Note. Boldface indicates highest factor loadings. **item is reversed. *Cursive indicates an English translation only for the sake of understanding.*

Table S3. Final 30 items and subscales

Action Cancellation	
FRIS_17	Auch wenn mir etwas schmeckt, höre ich auf zu essen, wenn ich satt bin.
FRIS_18**	Bei meinen Lieblingsgerichten esse ich über meinen Hunger hinaus.
FRIS_19	Sobald ich merke, dass ich satt bin, beende ich mein Essen.
FRIS_20	Wenn ich während des Essens merke, dass ich schon satt bin, höre ich auf zu essen.
FRIS_21	Ich nehme mir nur dann eine weitere Portion, wenn ich noch nicht satt bin.
FRIS_24	Wenn ich mir vornehme, nur wenig zu essen, halte ich das auch ein.
FRIS_30	Wenn Essen verfügbar ist, fällt es mir leicht, das Essen über mehrere Portionen oder Mahlzeiten einzuteilen.
Withholding in Social Situations	
FRIS_1**	In Situationen, in denen viel Essen verfügbar ist (z. B. Buffet, Feierlichkeiten, Grillen) greife ich zu, obwohl ich satt bin.
FRIS_3**	Wenn ich mit Menschen zusammen bin, die etwas essen, esse ich mit, ohne darüber nachzudenken.
FRIS_4**	Wenn ich eine Person sehe, die etwas isst, habe ich das Bedürfnis etwas zu essen und tue dies dann auch.
FRIS_5**	Wenn mir eine andere Person etwas zu essen anbietet, was mir schmeckt, greife ich direkt zu.
FRIS_9**	Wenn andere etwas von ihrem Essen übriglassen, esse ich die Reste auf, obwohl ich satt bin.
FRIS_22**	In Situationen, in denen viel Essen zur Verfügung steht (z. B. Buffet, Grillen, Feierlichkeiten), esse ich weiter, obwohl ich schon satt bin.
FRIS_27**	Solange andere noch essen, kann ich nicht aufhören zu essen, obwohl ich satt bin.
Resisting despite Craving	
FRIS_10**	Ich esse unbewusst zwischen den Mahlzeiten.
FRIS_12**	Obwohl ich bestimmte Lebensmittel nicht essen will, kann ich ihnen dennoch nicht widerstehen.
FRIS_14**	Wenn ich Heißhunger auf ein bestimmtes Essen verspüre, kann ich nicht widerstehen und greife zu.
FRIS_15**	Wenn ich Lust auf etwas habe, esse ich es.
FRIS_23**	Ich nasche bis die angefangene Packung (z. B. Schokolade, Chips, Gummibärchen oder ähnliches) aufgebraucht ist.
FRIS_31**	Wenn ich Hunger habe, wähle ich Nahrungsmittel, die sehr wenig Zubereitung erfordern, damit ich schnell essen kann.
FRIS_33**	Wenn ich hungrig bin, wähle ich schnell verfügbares Essen.
Withstanding Rewarding Food	
FRIS_34**	Essen stellt eine Belohnung für mich dar.
FRIS_35**	Die Erwartung an Essen stimmt mich sehr glücklich.
FRIS_36**	Ich belohne mich mit Essen.
FRIS_37**	Wenn ich esse, fühle ich mich gut.
FRIS_38**	Ich bevorzuge Nahrungsmittel, die mich schnell belohnen.
Action Withholding	
FRIS_6	Der Anblick von Nahrungsmitteln (z. B. im Fernsehen, auf Werbeplakaten, in Zeitschriften oder ähnliches) verleitet mich nicht zum Essen.
FRIS_7	Der Anblick von Nahrungsmitteln (z. B. im Supermarkt, beim Bäcker, auf Märkten, in Geschäften oder ähnliches) verleitet mich nicht zum Essen.
FRIS_8	Auch wenn ich Gelegenheit zum Naschen habe (z. B. beim Kochen, Backen oder ähnliches), tue ich dies nicht.
FRIS_13	Wenn ich nicht naschen will, mache ich es auch nicht.

** Item is reversed.

Table S4. Items dropped due to cross-or low-loading (<.30) to increase internal consistency

FRIS_2**	Ich esse nach einem Hauptgang, der mich sehr satt gemacht hat, noch ein Dessert.
FRIS_11	Ich plane meine Mahlzeiten und halte mich daran.
FRIS_16	Bei lang ausgedehnten Essen (z. B. Raclette, Fondue oder ähnliches) fällt es mir leicht, das Essen zu beenden, wenn ich satt bin.
FRIS_25	Wenn ich angefangen habe zu naschen, kann ich aufhören, wenn ich möchte.
FRIS_26**	Wenn ich angefangen habe zu essen, kann ich nicht mehr aufhören.
FRIS_28**	Wenn etwas frisch aus dem Ofen kommt (z. B. Kuchen, Auflauf oder Brot), kann ich nicht warten, bis es abgekühlt ist und esse es gleich.
FRIS_29	Wenn ich zum Essen eingeladen bin, verzichte ich vorher auf eine Mahlzeit, um dann mehr essen zu können.
FRIS_32**	Wenn ich Hunger habe, esse ich eine vorbereitete Mahlzeit lieber kalt als sie noch einmal aufzuwärmen.
FRIS_39	Ich verzichte auf bestimmte Nahrungsmittel, weil sie meinem Körper auf lange Sicht nicht guttun.
FRIS_40	Beim Essen fällt mir eher auf, was mir nicht schmeckt, als was mir schmeckt.

References Supporting Information

1. Costello A, Osborne J. Best Practices in Exploratory Factor Analysis: Four Recommendations for Getting the Most From Your Analysis. *Pract Assess Res Eval* 2005;10:1–9.
